# Supplementary material for: Targeting the High-Density Lipoprotein Proteome for the Treatment of Post-Acute Sequelae of SARS-CoV-2
Source: Int J Mol Sci. 2024 Apr 20;25(8):4522. doi: 10.3390/ijms25084522 (PMC11049911; doi:10.3390/ijms25084522)
Supplement: Supplementary file 1 [file ijms-25-04522-s001.zip › ijms-2893588-supplementary.pdf]

Supplemental Material

Targeting the High-density Lipoprotein Proteome for the Treatment of Postacute Sequelae of SARS-CoV-2

Karsten Grote[1†], Ann-Christin Schaefer[1†], Muhidien Soufi[1], Volker Ruppert[1], Uwe Linne[2], Aditya Bhagwat[3], Witold Szymanski[3], Johannes Graumann[3], Yana Gercke[1], Sümeya Aldudak[1], Denise Hilfiker-Kleiner[4], Elisabeth Schieffer[1†], Bernhard Schieffer[1†*]

[1] Department of Cardiology, Angiology, and Intensive Care; Philipps University Marburg, Marburg, 35043, Germany
[2] Mass Spectrometry Facility, Department of Chemistry; Philipps University Marburg, Marburg, 35043, Germany
[3] Institute of Translational Proteomics; Philipps University Marburg, Marburg, 35043, Germany
[4] Institute Cardiovascular Complications in Pregnancy and Oncologic Therapies; Philipps University Marburg, Marburg, 35043, Germany
* Correspondence: bernhard.schieffer@staff.uni-marburg.de
† These authors contributed equally to this work.

Supplementary Figures and Tables

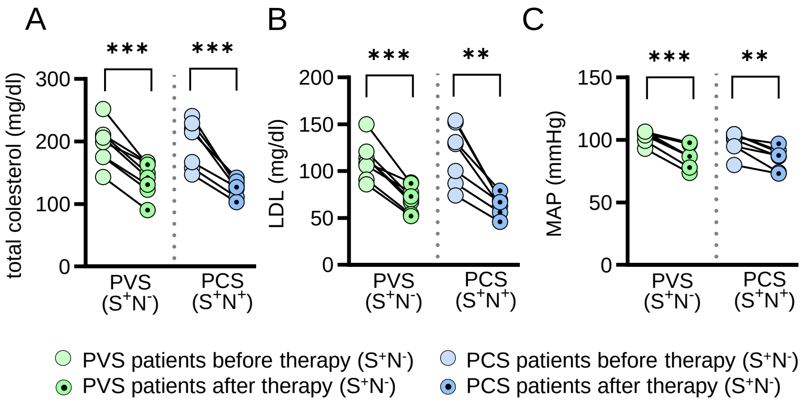

Supplemental Figure S1. Cholesterol and blood pressure before and after therapy in PVS and PCS patients. (**A**) total cholesterol, (**B**) LDL and (**C**) MAP. PVS = post-vaccination syndrome, PCS = post-COVID syndrome, S = spike protein, N = nucleocapsid protein, LDL = low-density lipoprotein, HDL = high-density lipoprotein, MAP = mean arterial pressure.

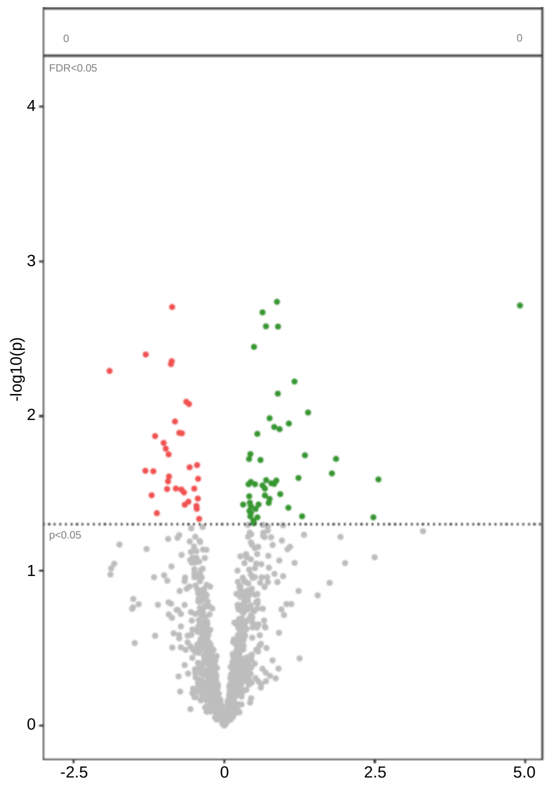

Supplemental Figure S2. No differences in protein abundance in the HDL proteome of controls and patients in relation to vaccinated vs. vaccinated with past infection. Protein abundance in the HDL of control and patients (PVS/PCS) were compared in regard to their antibody status ($S^{+}N^{-}$ vs. $S^{+}N^{+}$) and are presented as volcano plot. More abundant proteins are shown in green and less abundant proteins are shown in red. PVS = post-vaccination syndrome, PCS = post-COVID syndrome, S = spike protein, N = nucleocapsid protein, HDL = high-density lipoprotein.

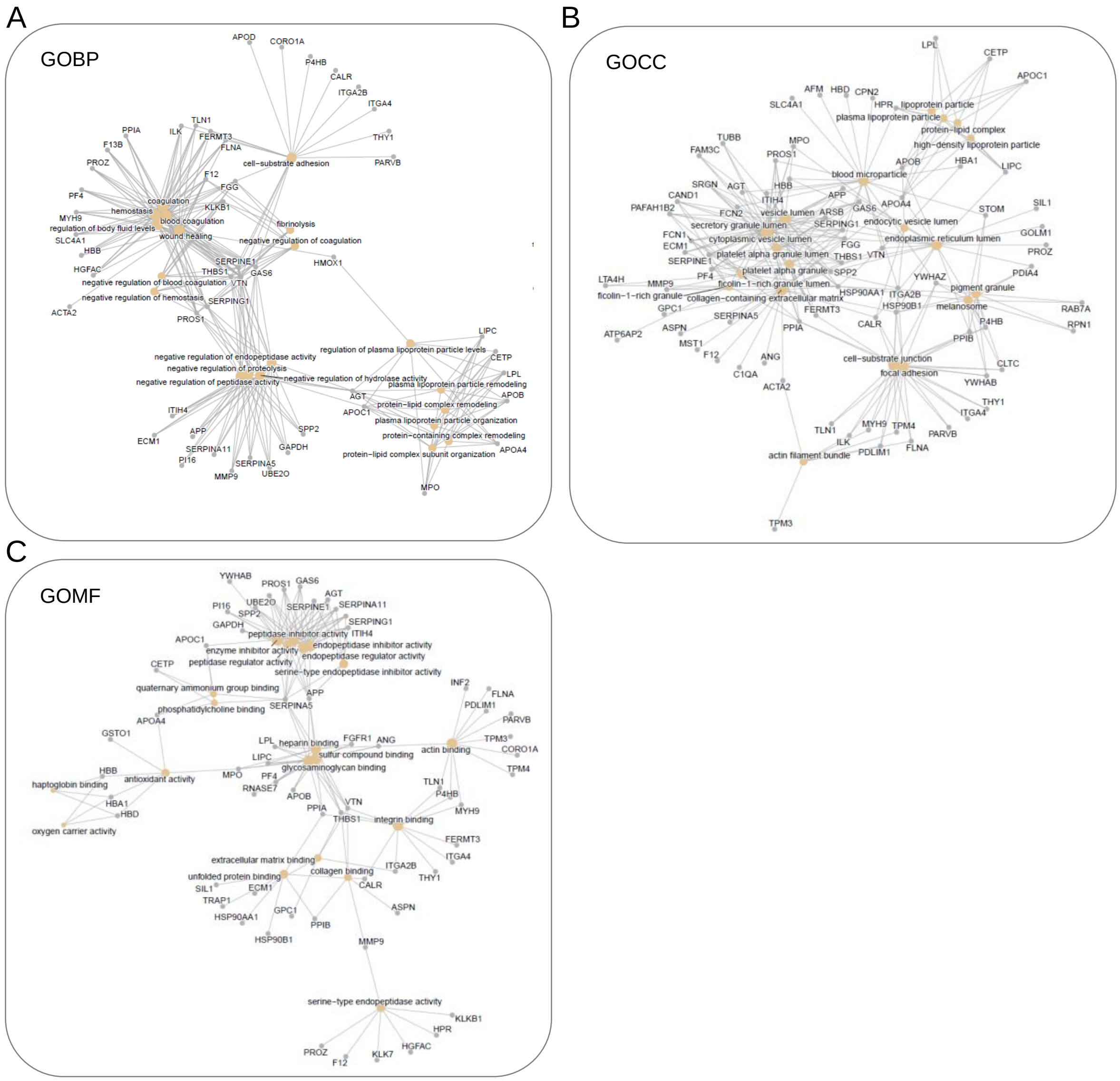

Supplemental Figure S3. Differentially abundant proteins ($p<0.05$) in the HDL proteome of PVS/PCS patients compared to controls are shown in a pathway enrichment analysis using (**A**) gene ontology biological process (GOBP), (**B**) gene ontology cellular component (GOCC) and (**C**) gene ontology molecular function (GOMF) annotations. PVS = post-vaccination syndrome, PCS = post-COVID syndrome, HDL = high-density lipoprotein.

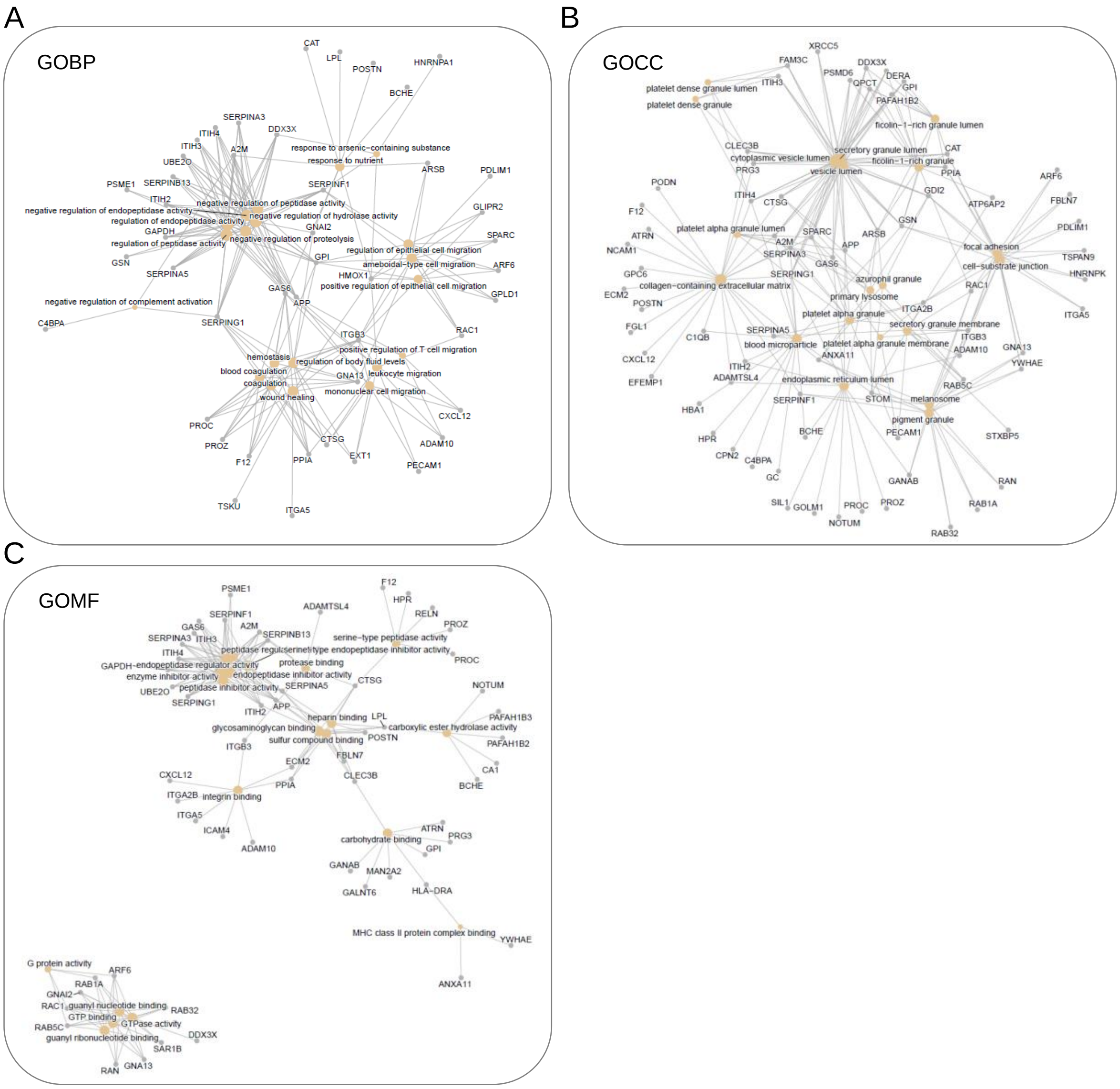

Supplemental Figure S4. Differentially abundant proteins (p<0.05) in the HDL proteome of PVS/PCS patients after therapy compared to before are shown in a pathway enrichment analysis using (**A**) gene ontology biological process (GOBP), (**B**) gene ontology cellular component (GOCC) and (**C**) gene ontology molecular function (GOMF) annotations. PVS = post-vaccination syndrome, PCS = post-COVID syndrome, HDL = high-density lipoprotein.

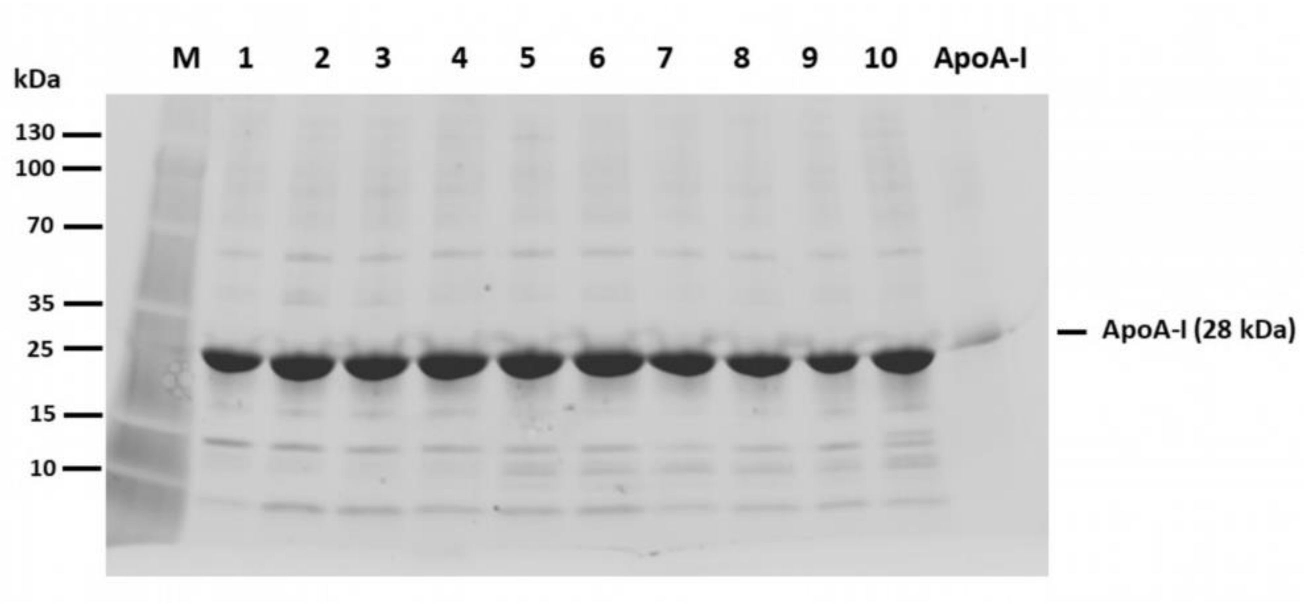

Supplemental Figure S5. SDS-PAGE of HDL samples. Inverse image of representative HDL isolates from serum samples subjected to 4-20% SDS-PAGE. 20 µg total protein was applied. The major band on the gels represents ApoA-I. Purified human ApoA-I was used as control and a protein ladder as a molecular weight standard (M). kDa = kilodalton. HDL = high-density lipoprotein.

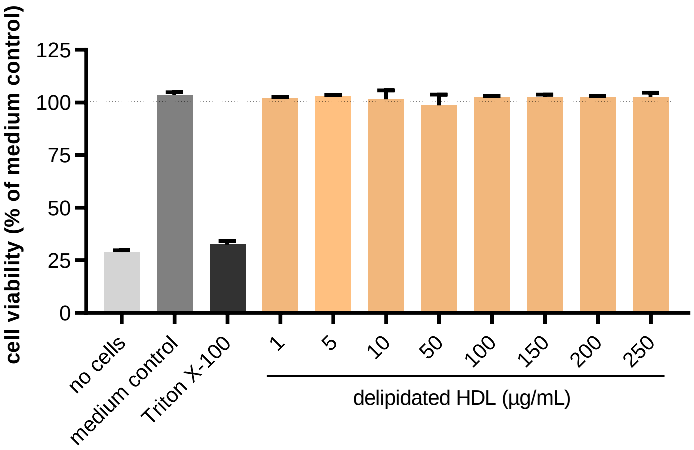

Supplemental Figure S6. Cell viability (%) of EAh.926 cells after 6 hrs of stimulation with delipidated HDL (1-250 µg/mL) was determined using alamarBlue cell viability assay. Conditions without cells and Triton X-100 were used as positive controls and cell culture medium as negative control. n = 2. HDL = high-density lipoprotein.

Supplemental Table S1. Symptoms of PVS and PCS patients.

	patients	
antigen-status	PVS (S^+/N^-) n=8	PCS (S^+/N^+) n=7
duration of symptoms (days)	319.5(107-376)	329.5(150-400)
muscle pain	5	4
headache	4	6
impairment of the ability to concentrate and remember	6	6
perceptual and sensory disorders	6	4
movement coordination disorder, muscle weakness, -twitching	6	4
reading disorders, word-finding disorders, information processing disorder	4	4
dizziness when standing up too quickly	1	4
dizziness and lightheadedness	3	7
impaired intestinal motility	6	4
shortness of breath at rest/light physical exertion	6	3
increase or loss of appetite, weight gain or weight loss	6	3
painful lymph nodes	4	1
palpitations, rapid pulse, heart stuttering	7	5
chest pain, chest tightness	5	4
brain fog	4	5
fatigue	5	6
restrictions in the social environment	8	5

Median and range are shown for duration of symptoms. Main symptoms reported (prevalence >50%). PVS = post-vaccination syndrome, PCS = post-COVID syndrome, S = spike protein, N = nucleocapsid protein.

Supplemental Table S2. Comorbidities in controls and PVS/PCS patients.

	control		patients	
antigen-status	S^+/N^- n=8	S^+/N^+ n=8	PVS (S^+/N^-) n=8	PVS (S^+/N^+) n=7
allergies present	3	2	6	3
Hashimoto Thyreoiditis	1	1	2	1
Migraine	-	1	2	1
Scoliosis	1	1	1	1
state after viral infection (EBV/CMV/Meningitis)	-	-	3	-
bronchial asthma	2	-	1	-
endometriosis	-	1	1	-
neurodermatitis	-	-	1	1
spinal stenosis	-	-	-	1
cranio mandibular dysfunction	-	-	1	-
herniated disc	-	-	1	-
condition after type b gastritis	-	-	-	-
diabetes mellitus I	-	-	1	-
hypothyreosis	-	-	1	-
chronic back pain	-	-	-	1
primary M. Raynaud	-	-	1	-
state after M. Hodgkin	-	-	1	-
condition after hemorrhagic shock with mass transfusion	-	-	-	1
state after cervical cancer	1	-	-	-
Morbus Meulengracht	1	-	-	-
benign paroxysmal positional vertigo	-	1	-	-
polyneuropathy	1	-	-	-
M. Basedow	1	-	-	-
arterial hypertension	1	-	-	-

PVS = post-vaccination syndrome, PCS = post-COVID syndrome, S = spike protein, N = nucleocapsid protein.

Supplemental Table S3. Medication of PVS and PCS patients.

	PVS patients	PCS patients
antigen-status	S^+/N^- (n=8)	S^+/N^+ (n=7)
statin		
Rosuvastatin (10 mg/day)	6	5
Simvastatin (20 mg/day)	2	1
Atorvastatin (10 mg/day)	-	1
ARB		
Candesartan (4 mg/day)	1	1
Candesartan (8 mg/day)	6	3
Candesartan (16 mg/day)	-	2
Telmisartan (20 mg/day)	-	1
Telmisartan (80 mg/day)	1	-

PVS = post-vaccination syndrome, PCS = post-COVID syndrome, S = spike protein, N = nucleocapsid protein. ARB = angiotensin II receptor blocker.

Supplemental Table S4. Blood pressure and cholesterol and its fractions inflammatory markers in healthy controls and PVS/PCS patients before and after therapy.

	control	PVS patients			control	PCS patients		
antigen-status	S^+/N^-	S^+/N^- (n=8)	S^+/N^- (n=8)		S^+/N^+	S^+/N^+ (n=7)	S^+/N^+ (n=7)	
therapy	no	before	after	P	no	before	after	P
systolic blood pressure (mmHg)	-	129 (108-138)	110 (96-129)	A -<br>B -<br>C *0.02*	-	122 (100-132)	110 (83-122)	A -<br>B -<br>C *0.008*
diastolic blood pressure (mmHg)	-	91 (84-99)	75 (59-85)	A -<br>B -<br>C *0.009*	-	89 (70-96)	76 (68-85)	A -<br>B -<br>C *0.0006*
MAP (mmHg)	-	105 (93-107)	87 (74-98)	A -<br>B -<br>C *0.00004*		100 (80-105)	88 (73-97)	A -<br>B -<br>C *0.002*
total cholesterol (mg/dl)	191.5 (154-399)	202.5 (143-252)	145.0 (90-167)	A 0.439<br>B *0.019*<br>C *0.00003*	180.5 (141-213)	215.0 (147-241)	127.0 (103-142)	A 0.283<br>B *0.0003*<br>C *0.0006*
LDL (mg/dl)	111.5 (89-255)	111.5 (86-150)	71.5 (52-88)	A 0.360<br>B *0.011*<br>C *0.00005*	109.0 (79-127)	129.0 (74-154)	67.0 (46-79)	A 0.363<br>B *0.0002*<br>C *0.001*
HDL (mg/dl)	58.0 (51-68)	63.5 (45-78)	62.5 (38-77)	A 0.328<br>B 0.786<br>C 0.056	52.5 (46-68)	53.0 (46-64)	51.0 (43-56)	A 0.804<br>B 0.272<br>C 0.200

Median and range are shown. PVS = post-vaccination syndrome, PCS = post-COVID syndrome, S = spike protein, N = nucleocapsid protein, LDL = low-density lipoprotein, HDL = high-density lipoprotein, MAP = mean arterial pressure. A = PVS/PCS patients before therapy vs. control, B = PVS/PCS patients after therapy vs. control, C = PVS/PCS patients after therapy vs. PVC/PCS patients before therapy.

Supplemental Table S5. Circulating inflammatory markers in healthy controls and PVS/PCS patients before and after therapy.

	Control	PVS patients			control	PCS patients		
antigen-status	S^+/N^-	S^+/N^- (n=8)	S^+/N^- (n=8)		S^+/N^+	S^+/N^+ (n=7)	S^+/N^+ (n=7)	
therapy	No	before	after	P	no	before	after	P
leucocytes (G/l)	6.3	7.3	6.8	A 0.326	6.5	5.4	5.1	A 0.398
	(4.5-8.7)	(5.0-11.3)	(5.8-10.7)	B 0.458	(3.5-7.1)	(4.1-6.7)	(4.3-6.1)	B 0.064
				C 0.593				C 0.159
lymphocytes (G/l)	1.9	1.7	1.9	A 0.709	1.8	1.6	1.7	A 0.246
	(1.2-3.0)	(1.1-2.9)	(1.2-3.0)	B 0.876	(1.1-2.7)	(1.2-1.9)	(1.4-2.1)	B 0.395
				C 0.090				C 0.329
monocytes (%)	8.8	6.9	6.9	A 0.309	6.3	8.4	8.4	A 0.193
	(4.4-9.6)	(5.2-10.2)	(5.1-9.4)	B 0.275	(5.0-11.0)	(6.6-11.2)	(7.3-10.1)	B 0.077
				C 0.906				C 0.470
CRP (mg/dl)	1.1	1.2	1.3	A 0.659	1.5	0.7	0.4	A 0.197
	(0.2-10.8)	(0.2-6.2)	(0.3-7.2)	B 0.883	(0.2-6.5)	(0.5-2.4)	(0.2-2.1)	B 0.100
				C 0.273				C *0.003*
IL-1 (ng/l)	5.0	5.0	5.0	A 0.214	5.0	5.0	5.0	A 0.335
	(5.0-22.1)	(5.0-8.7)	(5.0-5.0)	B 0.182	(5.0-6.6)	(5.0-5.6)	(5.0-5.5)	B 0.272
				C 0.373				C 0.830
sIL-2R (kU/l)	346.0	438.0	422.5	A 0.426	467.5	417.0	448.0	A 0.748
	(265-648)	(330-647)	(370-698)	B 0.194	(275-680)	(406-515)	(302-1155)	B 0.606
				C 0.761				C 0.496
IL-6 (pg/ml)	1.5	2.0	1.8	A 0.301	1.5	2.0	2.0	A 0.511
	(1.5-3.0)	(2.0-8.0)	(1.5-6.0)	B 0.534	(1.5-6.0)	(1.5-2.0)	(1.5-3.0)	B 0.747
				C 0.067				C 0.407
IL-8 (ng/l)	6.7	8.0	10.3	A 0.190	6.9	7.0	8.8	A 0.441
	(5.0-8.1)	(5.0-13.2)	(6.3-13.3)	B 0.056	(5.0-35.9)	(5.0-10.4)	(5.0-18.1)	B 0.818
				C 0.231				C 0.380
IL-10 (ng/l)	5.0	5.0	5.0	A 0.453	5.0	5.0	5.0	A 0.300
	(5.0-5.0)	(5.0-6.9)	(5.0-5.0)	B 0.297	(5.0-5.0)	(5.0-11.4)	(5.0-14.0)	B 0.300
				C 0.363				C 0.373
TNF-α (ng/l)	6.1	5.0	7.3	A 0.288	6.0	6.2	7.0	A 0.716
	(5.3-9.8)	(4.0-8.1)	(4.0-9.8)	B 0.952	(4.8-7.8)	(4.2-6.9)	(5.5-11.2)	B 0.209
				C 0.872				C 0.905

Median and range are shown. PVS = post-vaccination syndrome, PCS = post-COVID syndrome, S = spike protein, N = nucleocapsid protein, CRP = c-reactive protein, IL = interleukin, sIL-2R = soluble interleukin 2 receptor, TNF = tumor necrosis factor. P-values: A = PVS/PCS patients before therapy vs. control, B = PVS/PCS patients after therapy vs. control, C = PVS/PCS patients after therapy vs. PVC/PCS patients before therapy.

Supplemental Table S6. Medication and changes in total cholesterol, LDL, HDL, MAP and scoring of PVS and PCS patients.

		change (mg/dl)			change (mmHg)	improvement/deterioration (scoring)			
antigen-status	statin	chol.	LDL	ARB	MAP	Bell	CFQ	FAS	SF-36
PVS patients S+/N- (n=8)	Rosuvastatin (10 mg/day)	-52	-47	Candesartan (8 mg/day)	-8.3	+	+	+	+
	Rosuvastatin (10 mg/day)	-44	-21	Candesartan (8 mg/day)	-17.7	+	+	+	+
	Rosuvastatin (10 mg/day)	-33	-29	Candesartan (8 mg/day)	-19.3	+	+	+	+
	Rosuvastatin (10 mg/day)	-62	-44	Candesartan (8 mg/day)	-18.3	+	+	o	+
	Rosuvastatin (10 mg/day)	-89	-63	Candesartan (8 mg/day)	-21.3	+	+	o	+
	Rosuvastatin (10 mg/day)	-64	-48	Telmisartan (80 mg/day)	-8.7	+	+	+	+
	Simvastatin (20 mg/day)	-45	-37	Candesartan (4 mg/day)	-17.0	+	+	-	+
	Simvastatin (20 mg/day)	-53	-34	Candesartan (8 mg/day)	-20.0	+	+	+	+
PCS patients S+/N+ (n=7)	Rosuvastatin (10 mg/day)	-96	-66	Candesartan (4 mg/day)	-13.7	+	+	+	+
	Rosuvastatin (10 mg/day)	-112	-83	Candesartan (8 mg/day)	-13.3	+	+	+	+
	Rosuvastatin (10 mg/day)	-73	-52	Candesartan (8 mg/day)	-5.0	+	+	+	+
	Rosuvastatin (10 mg/day)	-93	-85	Candesartan (16 mg/day)	-14.7	+	+	+	+
	Rosuvastatin (10 mg/day)	-47	-31	Candesartan (16 mg/day)	-21.0	+	+	+	+
	Simvastatin (20 mg/day)	-44	-28	Candesartan (8 mg/day)	-7.3	+	+	+	+
	Atorvastatin (10 mg/day)	-40	-33	Telmisartan (20 mg/day)	-7.0	+	+	+	+

PVS = post-vaccination syndrome, PCS = post-COVID syndrome, S = spike protein, N = nucleocapsid protein, chol. = total cholesterol, LDL = low-density lipoprotein, ARB = angiotensin II receptor blocker, MAP = mean arterial pressure, Bell = Bell disability score, CFQ = Chalder fatigue scale, FAS = fatigue assessment scale, SF-36 = SF-36 physical functioning sum scale, + = improvement, - = deterioration, o = no change.

Supplemental Table S7. Significantly altered proteins in the HDL proteome of PVS/PCS patients vs. controls.

acronym	full name	Uniprot ID	gene ID	log2 (FC)	FDR	HDL prot.	kDa
	enhanced						
MYL6	myosin light polypeptide 6	P60660	4637	2.27	0.001	+	16.9
APP	amyloid-beta precursor protein	P05067	351	1.38	0.002	-	86.9
TLN1	talin-1	Q9Y490	7094	1.70	0.002	+*	26.9
PPIA	peptidyl-prolyl cis-trans isomerase A	P62937	5478	1.37	0.003	+	18.0
HBB	hemoglobin subunit beta	P68871	3043	1.53	0.006	+*	15.9
HGFA	hepatocyte growth factor activator	Q04756	3083	1.09	0.006	+	70.6
PARVB	beta-parvin	Q9HBI1	29780	1.85	0.006	+*	41.7
TPM4	tropomyosin alpha-4 chain	P67936	7171	1.32	0.006	+	28.5
MAN1A1	mannosyl-oligosaccharide 1,2-alpha-mannosidase IA	P33908	4121	1.08	0.006	-	72.9
FERMT3	FERM domain containing kindlin 3	Q86UX7	83706	1.20	0.006	+*	75.9
HBD	hemoglobin subunit delta	P02042	3045	1.53	0.009	+	16.0
MST1	hepatocyte growth factor-like protein	P26927	4485	1.22	0.012	-	80.3
TRAP1	heat shock protein 75 kDa	Q12931	10131	2.04	0.017	-	80.1
FLNA	filamin-A	P21333	2316	1.14	0.017	+*	280.7
PIBF1	progesterone-induced-blocking factor 1	Q8WXW3	10464	1.73	0.018	+	89.8
HBA1	hemoglobin subunit alpha	P69905	3039	1.47	0.026	+*	15.2
PROZ	vitamin K-dependent protein Z	P22891	8858	1.20	0.028	+	44.7
NAPA	alpha-soluble NSF attachment protein	P54920	8775	0.86	0.031	-	33.2
HSP90B1	heat shock protein 90 beta family member 1	P14625	7184	0.91	0.031	+	92.4
SERPINA11	serpin A11	Q86U17	256394	0.83	0.041	+*	46.9
	reduced						
PF4;PF4V1	platelet factor 4	P02776; P10720	5196	-2.66	0.001	-	10.8, 11.5
NT5DC3	5'-nucleotidase domain-containing protein 3	Q86UY8	51559	-2.06	0.002	-	63.4
PAFAH1B2	platelet-activating factor acetylhydrolase IB subunit alpha2	P68402	5049	-3.55	0.006	-	25.5
ATP6AP2	ATPase H^+ transporting accessory protein 2	O75787	10159	-0.97	0.029	-	39.0
ARSB	arylsulfatase B	P15848	411	-0.67	0.031	-	59.6
ITIH4	inter-alpha-trypsin inhibitor heavy chain H4	Q14624	3700	-0.62	0.044	+*	103.3

PVS = post-vaccination syndrome, PCS = post-COVID syndrome, FC = fold-change, FDR = false discovery rate, HDL prot. = high-density lipoprotein proteome watch **+***= detected in at least 3 different studies (HDL proteome watch).

Supplemental Table S8. Significantly altered proteins in the HDL proteome of PVS/PCS patients after vs. before therapy.

acronym	full name	Uniprot ID	gene ID	log2 (FC)	FDR	HDL prot.	kDa
enhanced							
FAM3C	FAM3 metabolism-regulating signaling molecule C	Q92520	10447	2.00	0.029	-	24.6
ATP6AP2	ATPase H^+ transporting accessory protein 2	O75787	10159	1.15	0.029	-	39.0
ADAM10	disintegrin and metalloproteinase domain-containing protein 10	O14672	102	1.00	0.034	-	84.1

PCS = post-COVID syndrome, FC = fold-change, FDR = false discovery rate, HDL prot. = high-density lipoprotein proteome watch.

Supplemental Table S9. Summary of significantly altered proteins in the HDL proteome of PVS/PCS patients vs. controls and of PVS/PCS patients after vs. before therapy.

PVS/PCS patients vs. control		PVS/PCS patients after therapy vs. PVS/PCS patients before therapy
enhanced	*reduced*	*enhanced*
MYL6	PF4;PF4V1	FAM3C
APP	NT5DC3	**ATP6AP2**
TLN1	PAFAH1B2	ADAM10
PPIA	**ATP6AP2**	
HBB	ARSB	
HGFA	ITIH4	
PARVB		
TPM4		
MAN1A1		
FERMT3		
HBD		
MST1		
TRAP1		
FLNA		
PIBF1		
HBA1		
PROZ		
NAPA		
HSP90B1		
SERPINA11		
